# TriNetX and Real-World Evidence: A Critical Review of Its Strengths, Limitations, and Bias Considerations in Clinical Research

**DOI:** 10.71079/aside.im.03222516

**Published:** 2025-03-22

**Authors:** Mahmoud Nassar, Hazem Abosheaishaa, Khaled Elfert, Azizullah Beran, Abdellatif Ismail, Mouhand Mohamed, Anoop Misra, Muhammed Amir Essibayi, David J. Altschul, Ahmed Y. Azzam

**Affiliations:** 1 Department of Medicine, Jacobs School of Medicine and Biomedical Sciences, University at Buffalo, Buffalo, NY, USA.; 2 Internal Medicine Department, Icahn School of Medicine at Mount Sinai, NYC H+H Queens, New York, NY, USA.; 3 Division of Gastroenterology, West Virginia University School of Medicine, Morgantown, WV, USA.; 4 Division of Gastroenterology and Hepatology, Indiana University, Indianapolis, IN, USA.; 5 Department of Internal Medicine, University of Maryland Medical Center Midtown, Baltimore, MD, USA.; 6 Division of Gastroenterology and Hepatology, May Clinic, Rochester, MN, USA.; 7 National Diabetes, Obesity and Cholesterol Foundation (N-DOC), New Delhi, Delhi, India.; 8 Department of Neurological Surgery, Montefiore Medical Center, Albert Einstein College of Medicine, Bronx, NY, USA.; 9 Montefiore-Einstein Cerebrovascular Research Lab, Montefiore Medical Center, Albert Einstein College of Medicine, Bronx, NY, USA.

**Keywords:** TriNetX, Clinical Research, Data Quality, Real-World Evidence, Research Methodology

## Abstract

**Introduction:**

The increasing utilization of real-world data platforms in medical research necessitates a comprehensive understanding of their methodological strengths and limitations. TriNetX has emerged as a significant platform for exploring large healthcare datasets. This review aims to critically evaluate the methodological framework and limitations of TriNetX, assess the impact of electronic health record coding accuracy on data reliability, and analyze the platform’s capacity for generating generalizable real-world evidence in clinical research.

**Methods:**

We conducted a comprehensive review examining TriNetX’s data architecture, quality metrics, and research applications, focusing on data integrity, platform architecture, and the external validity of research findings.

**Results:**

The analysis reveals significant methodological considerations. TriNetX’s reliance on retrospective data introduces biases such as selection bias and confounding variables. The coding accuracy of electronic health records, which have not been independently validated, is a critical determinant of data reliability. The demographic representation is limited, affecting the generalizability of results.

**Discussion:**

Despite its extensive use, TriNetX’s effective utilization requires careful consideration of its inherent limitations. The platform’s data, predominantly from insured populations in academic and acute care settings, may not fully represent broader demographic groups. Addressing these methodological constraints is crucial for enhancing the reliability and applicability of research findings derived from TriNetX.

**Conclusions:**

TriNetX is a valuable resource for healthcare research. However, its limitations must be acknowledged, and future research should focus on standardizing data collection and enhancing data validation processes to mitigate platform-specific biases and improve the quality and applicability of the findings.

## 1. Introduction

The increasing reliance on real-world data platforms in medical research has transformed the landscape of clinical evidence generation. Among these platforms, TriNetX has emerged as a significant player, providing real-time access to anonymized electronic medical records (EMR) and claims data from millions of patients globally [1]. While several studies have utilized TriNetX for large-scale observational research, a comprehensive systematic evaluation of its methodological framework, limitations, and research applications remains notably absent from the literature [2].

Current knowledge gaps in real-world data platforms center around three critical areas that demand systematic investigation. These encompass the impact of data quality variations on research outcomes, the methodological considerations in managing platform-specific biases, and the external validity of findings derived from such platforms [3]. Despite the growing adoption of TriNetX in medical research, existing literature has primarily focused on individual study outcomes rather than systematically evaluating the platform’s capabilities and limitations. This gap is particularly significant given the platform’s increasing influence on clinical decision-making and research methodology [4].

The novelty of our systematic review lies in its comprehensive review of TriNetX’s architecture, data quality metrics, and research applications through methodological lens. Unlike previous platform-specific analyses, which have typically focused on individual aspects such as data completeness or specific disease outcomes, our review provides an integrated assessment of the entire research ecosystem. This approach enables a deeper understanding of how various components from data capture to analysis interact and influence research outcomes [5–8].

TriNetX has revolutionized evidence-based medical research through its extensive network of healthcare systems. The platform facilitates rapid cohort identification and supports comprehensive research capabilities, including instant dataset queries for patient demographics, comorbidities, and treatment histories. This technological framework enhances study design efficiency and execution while implementing advanced analytical tools, such as propensity score matching, to minimize bias in observational studies and improve finding reliability [9–11].

However, the platform’s dependence on aggregated, real-world data presents significant methodological challenges that warrant careful consideration. The integrity of research outcomes may be compromised by potential issues in data completeness and accuracy, as information is sourced from EMR and claims data that may contain inherent errors or inconsistencies [12, 13]. These challenges are further complicated by variations in coding practices, documentation standards, and data collection methods across healthcare systems [1, 14, 15]. While TriNetX offers sophisticated analytical tools, observational studies remain vulnerable to unmeasured confounding, limiting causal inference compared to randomized controlled trials. Furthermore, the representativeness of the data warrants careful consideration, as it reflects specific patient populations that may not adequately represent broader demographic groups [16, 17].

This systematic review addresses three fundamental objectives in understanding and optimizing TriNetX’s research applications. First, it provides a comprehensive evaluation of the platform’s methodological framework and its implications for research quality. Second, it assesses the impact of data quality variations, coding practices, and technological disparities on research outcomes. Third, it establishes evidence-based recommendations for optimizing the platform’s utilization in medical research.

Our review fills critical gaps in the understanding of real-world data platforms through several innovative approaches. We systematically analyze the methodological considerations unique to TriNetX-based research while evaluating the platform’s capacity for generating reliable and generalizable evidence. Additionally, we provide a framework for assessing and mitigating platform-specific limitations and developing standardized approaches for managing data quality variations and potential biases. Through this comprehensive analysis, we aim to enhance researchers’ ability to effectively utilize TriNetX while maintaining methodological rigor and ensuring the validity of their findings in clinical research.

## 2. Methods:

### 2.1. Review Approach and Scope

We aimed to review the TriNetX platform’s capabilities, limitations, and implications for medical research. Our study focused on understanding the platform’s architecture, data quality considerations, and its application in generating real-world evidence. We conducted a review of the platform’s features, technical specifications, and documented research applications to provide a thorough understanding of its utility and limitations in healthcare research.

### 2.2. Thematic Framework

The organization of this review follows a thematic structure derived from critical aspects of research platform evaluation. Our analysis framework emerged from examining key operational and methodological aspects of TriNetX, including data collection processes, platform architecture, and research applications. The primary themes were developed to address fundamental aspects of the platform: inherent limitations of retrospective studies, EMR coding accuracy, diagnostic coding precision, platform-specific data limitations, network representativeness, risk of bias, technological disparities, practice variations, and external validity considerations.

### 2.3. Analysis Structure

Our review begins with a review of the inherent limitations of retrospective observational studies, providing context for understanding TriNetX’s operational framework. We then progress through interconnected themes, analyzing EMR coding accuracy and its implications for data reliability. The review extends to examine specific coding impacts on TriNetX, platform data limitations, and network representativeness. We further explore the risk of bias considerations, technological disparities, and practice variations that affect research outcomes. The analysis concludes with an assessment of external validity and recommendations for optimal platform utilization.

### 2.4. Synthesis Approach

The review synthesizes and highlights the current understanding of TriNetX’s capabilities and limitations, drawing from published literature and platform documentation. We examine how various methodological aspects interact and influence research outcomes, providing a view of the platform’s utility in medical research. This synthesis aims to guide researchers in effectively utilizing TriNetX while maintaining awareness of its methodological constraints and opportunities for optimization.

## 3. Discussion

### 3.1. Inherent limitations of retrospective observational studies

Susceptibility to bias in retrospective studies is considerable due to the fact that exposure and outcome data have already occurred and researchers cannot control participant selection, leading to potential selection bias [18, 19]. Additionally, the inability to randomize subjects leaves these studies vulnerable to confounding factors that may obscure the true relationship between variables, complicating causal inferences [20, 21]. Data quality is another critical issue, as the information is often collected for purposes other than research, like medical billing, resulting in data that may be incomplete, inconsistent, or inaccurate, thus affecting the reliability of the study [22, 23]. Recall bias further complicates matters, as patient-reported outcomes or historical records may be skewed by imperfect recollection or subjective interpretation [24, 25]. Temporal ambiguity in these studies often makes it challenging to establish a clear sequence between exposure and outcome, thereby hindering causality determination [26, 27]. Since retrospective studies depend on existing data, researchers have limited control over variables and cannot adjust for all relevant factors or unforeseen confounders [18, 28]. The representativeness of the study population may also not mirror the general population, limiting the generalizability of the findings [29, 30]. Despite the minimal harm to participants from using existing data, ethical considerations such as data privacy, consent, and the use of sensitive information still pose significant concerns [31, 32].

### 3.2. Accuracy of EMR coding

Issues surrounding the accuracy of EMR coding are multifaceted, reflecting significant challenges across various aspects of the coding process. Studies like that by Horsky et al. (2017) have demonstrated considerable variability in coding, often resulting in omissions or incorrect entries, with only half of the entered codes for specific diagnoses being appropriate and a high omission rate for secondary diagnoses such as nicotine dependence or dialysis dependence [33]. Automated coding tools, such as the TOLBIAC control for ICD-10 codes, show moderate accuracy (micro-average F-measure of 0.76 for drug prescriptions) but require enhancements in text analysis capabilities [34]. Reliance on billing codes can lead to substantial false positives, as evidenced by a study identifying lung cancer patients with an ICD-based method achieving only 65% precision [35]. Comparatively, automated systems have proven completer and more accurate than manual coding, highlighted by their superior performance in coding PQRI quality measures [36]. Accuracy also varies between specialists and generalists, with problem lists in EHRs being more accurate when managed by primary care providers than specialists [37]. Phenotyping challenges are significant, such as in chronic rhinosinusitis, where reliance solely on billing codes results in low precision and necessitates additional clinical validation [38]. Administrative coding errors are prevalent too; an audit of emergency medical admissions revealed changes in the primary diagnosis in over 16% of cases, impacting outcome metrics like morbidity indices [39]. The quality of ICD-10 coding heavily depends on the experience of the coders, as shown in initiatives to improve morbidity and mortality data accuracy in Lagos hospitals [40]. AI-based methods like adversarial autoencoders have been proposed to address issues of missing or incomplete clinical codes, demonstrating improvements in predictive performance [41]. Furthermore, big data concerns, such as inconsistencies in administrative coding for conditions like hospital-acquired venous thromboembolism, underscore the need for more robust diagnostic validation methods [42].

### 3.3. Errors in diagnostic coding

The impact of diagnostic coding errors on research outcomes is profound and multifaceted. Schrodi (2017) highlighted that errors in diagnostic coding could lead to misclassification of patient conditions, resulting in biased results in epidemiological and clinical studies, particularly diminishing the statistical power in genetic association studies, which underscores the critical need for accurate diagnostic code utilization [43]. Farzandipour and Sheikhtaheri (2009) through a systematic review, revealed that inaccuracies in ICD-10 codes significantly affect the validity of diagnoses in health databases, impacting both research findings and administrative decisions [44]. Usher et al. (2018) found that diagnostic coding errors during inter-hospital transfers are associated with increased inpatient mortality, emphasizing the necessity for robust health information exchange systems to enhance diagnostic accuracy [45]. Zafirah et al. (2018) noted that coding inaccuracies could lead to significant financial consequences by misassigning Diagnosis-Related Groups (DRGs), resulting in substantial revenue losses for hospitals [46]. Lorence and Ibrahim (2003) observed that studies using unvalidated diagnostic codes often lack reliability, with up to 87% of medical records in some datasets containing errors significant enough to alter research findings [47]. Diagnostic errors also complicate epidemiological research, as they can misrepresent disease prevalence and associated outcomes, necessitating validation studies to ascertain true associations [48]. Furthermore, Nouraei et al. (2015) discussed how variability in coding accuracy across healthcare facilities complicates the benchmarking of patient outcomes, affecting the comparability and utility of large datasets for outcomes management [39]. In **Table 1** we provided a review of the classification of diagnostic coding errors commonly encountered in EMR systems, their implications for research outcomes, and possible mitigation strategies.

### 3.4. Coding Impact Analysis on TriNetX

The analysis of EMR and ICD-10 coding issues affecting TriNetX reveals several key challenges. Palestine et al. (2018) noted imprecision in how ICD-10 codes are applied across different EMR systems, leading to inconsistencies in large-scale data analysis, particularly highlighting disparities in coding diseases like uveitis, which potentially introduces bias into research outcomes derived from pooled EHR data [49]. Kortüm et al. (2016) discussed the increased complexity post-EHR implementation, where the introduction of EMR systems with ICD-10 coding led to increased diagnosis diversity but also exposed suboptimal coding precision, affecting the accuracy and utility of clinical research databases like TriNetX. Stewart et al. (2019) found that transitioning from ICD-9 to ICD-10 disrupted the recording of specific mental health disorders due to mismatches in diagnostic coding frameworks, which could impact longitudinal studies utilizing databases such as TriNetX where such transitions lead to data inconsistencies [50]. Quan et al. (2005) highlighted challenges in comorbidity analysis, as coding algorithms for defining comorbidities in ICD-10 differ in their accuracy compared to previous ICD-9 models, influencing the quality of comorbidity data within databases like TriNetX and impacting their predictive utility [51]. Caskey et al. (2013) pointed out that misclassification and information loss are common when mapping ICD-9 codes to ICD-10, with up to 26% of pediatric diagnosis codes categorized as convoluted, posing challenges to the reliability of TriNetX data in pediatric research [52]. Additionally, Horsky et al. (2017) emphasized that high variation in coding precision across institutions due to human and system factors leads to inconsistencies in databases, complicating the interpretation of multicenter data like those aggregated in TriNetX [33].

### 3.5. Data Limitation in the Platform

One major limitation is the restricted data scope, as critical clinical and demographic details such as treatment adherence, lifestyle factors, and social determinants of health are often not captured. These omissions can lead to incomplete analyses and potential biases in understanding patient outcomes. Additionally, the relatively short observation periods inherent in the database further constrain its utility for studying long-term outcomes. Patients who leave the institution, relocate, or otherwise fall out of the network are no longer tracked, leading to gaps in longitudinal data and potential underestimation of adverse events or delayed outcomes. These limitations necessitate cautious interpretation of findings and, when possible, supplementation with external data sources to ensure more robust and generalizable conclusions.

### 3.6. Network Representativeness

Considerations of the representativeness of the TriNetX network reveal several limitations affecting the breadth and validity of its research outcomes. Topaloglu and Palchuk (2018) noted that although TriNetX connects healthcare organizations worldwide, its representativeness is primarily influenced by the geographic and demographic composition of its network, initially dominated by patients from the United States with scant representation from low- and middle-income countries, restricting the generalizability of research findings to global populations [4]. Despite significant growth in international representation, as TriNetX expanded from 55 healthcare organizations in seven countries in 2017 to over 220 organizations in 30 countries by 2022, disparities in data representation persist, impacting the validity of cross-regional comparisons [1]. Furthermore, including healthcare organizations with specific specialties or academic affiliations can skew the dataset towards populations frequently treated in those settings, such as cancer centers, limiting generalizability for broader disease populations [4]. González et al. (2020) highlighted that variability in data harmonization across institutions might lead to inconsistent quality, impacting the representativeness and reliability of aggregated data for multicenter research [53]. Moreover, Haudenschild et al. (2021) pointed out that the self-selection of participating healthcare organizations and reliance on EMR for data may introduce biases that disproportionately represent urban and technologically advanced healthcare systems, raising concerns about selection bias [54].

### 3.7. Risk of Bias

An in-depth examination of selection bias and unaccounted confounding factors presents significant challenges across various research designs. Cohort studies may show selection bias when inclusion criteria are linked to both exposure and outcomes, skewing results as higher hazard rates during early cohort inclusion periods suggest significant bias [55]. In Mendelian randomization, bias can arise from colliders—effects influenced by both genetic variants and confounders—with large selection effects amplifying this bias in simulations [56]. Unaccounted confounders in observational research can obscure causal relationships when variables influence both exposure and outcome [57]. Notably, selection bias impacts a study’s external validity, while confounding affects internal validity, crucial for comparative effectiveness research [58]. Missing data related to exposure or outcomes can also introduce bias, mitigated by techniques such as inverse probability weighting [59]. In environmental studies, selection bias in case-crossover designs occurs when reference periods don’t accurately represent exposure times, exacerbating bias [60]. Conditioning on colliders can create spurious associations in observational research [61], and in longitudinal studies, loss to follow-up can lead to bias if dropouts differ systematically from those who remain, with causal diagrams aiding in bias mitigation [62].

### 3.8. Technological Disparities

A discussion of technological disparities among hospitals reveals significant variations in access and utilization based on racial, ethnic, and regional differences, which in turn impact health outcomes. Kim et al. (2010) found that racial and ethnic minorities, such as Hispanic patients, are significantly less likely to utilize hospitals with advanced technological services like MRI and trauma units compared to white patients, underscoring systemic inequities [63]. Groeneveld et al. (2005) reported that hospitals serving higher proportions of minority populations often lag in adopting new medical technologies, contributing to racial gaps in access to advanced procedures such as coronary artery bypass grafting [64]. Sequist (2011) noted that while Health Information Technology (HIT) has the potential to reduce disparities, it is less accessible to safety-net hospitals, perpetuating inequities in the quality of care among underserved populations [65]. Economic constraints also play a critical role; according to David & Jahnke (2018), smaller or rural hospitals often face financial barriers to investing in new technologies, leading to disparities in equipment availability and maintenance compared to urban institutions [66]. Newton et al. (2010) highlighted that hospitals in low-resource settings struggle with challenges such as poorly trained staff and inadequate infrastructure, further widening the technological divide and compromising patient safety [67]. Lastly, Walker et al. (2020) observed disparities in the adoption of digital health tools like patient portals, with older and African American patients utilizing these technologies less frequently, indicating a digital divide even within technologically equipped hospitals [68].

### 3.9. Practice Disparities

Healthcare disparities and practices significantly limit the generalizability and applicability of research outcomes. Chin et al. (2012) identified that disparities in healthcare access and quality create heterogeneity in patient populations, leading to research outcomes that may not be generalizable across diverse groups, particularly when studies fail to account for racial and ethnic disparities [69]. Kilbourne et al. (2006) noted that research often focuses on settings with adequate resources, excluding under-resourced healthcare facilities and their patient populations, thus introducing selection bias and restricting the relevance of findings to broader, diverse settings [70]. Rust & Cooper (2007) highlighted that studies neglecting socioeconomic determinants of health, such as poverty or education, fail to provide actionable insights for reducing health disparities, further limiting the practical applicability of research[71]. Chinman et al. (2017) pointed out that research focusing narrowly on single interventions without considering multilevel determinants often falls short in addressing complex healthcare disparities, limiting the applicability of findings in real-world scenarios [72]. Koh et al. (2010) argued that research not tailored to cultural or linguistic contexts may result in ineffective interventions when applied to populations with specific needs, such as non-English speakers [73]. Lane et al. (2016) discussed how disparities in healthcare practices, such as differences in provider training and resource availability, complicate the translation of research into practice, reducing the impact of evidence-based interventions [74]. Dankwa-Mullan et al. (2010) observed that studies conducted in resource-rich environments often overlook the challenges faced by underfunded facilities, making their recommendations impractical for these settings [75]. Finally, Baker et al. (2001) emphasized that research lacking community engagement fails to address local needs and contexts, reducing the effectiveness of interventions when applied in diverse settings [76].

### 3.10. External Validity Critique

Critiquing the external validity and applicability of TriNetX study findings highlights several limitations in generalizing results to the general population. Stuart et al. (2015) noted that studies utilizing datasets might face challenges in population representativeness due to variability in patient demographics and institutional contributions, often not reflecting real-world diversity, which can limit generalizability[77]. Murad et al. (2018) found that the generalizability of findings is further complicated by differences in coding practices, healthcare delivery models, and patient characteristics among contributing healthcare organizations, leading to inconsistent applicability of results [78]. Kennedy-Martin et al. (2015) pointed out that many datasets exclude elderly patients or those with multiple comorbidities, which restricts the ability to generalize results to these high-need groups, affecting external validity [78]. Pearl & Bareinboim (2014) discussed how the inherent structure of datasets, relying on real-world data from diverse settings, can introduce selection bias and complicate causal interpretations, further impacting generalizability [79]. Leviton (2017) highlighted that interventions tested in highly specialized or resource-intensive institutions contributing to large databases may not be practical for low-resource or rural healthcare settings, limiting the utility of such findings [80]. Ling et al. (2023) mentioned that applying study results to populations not represented in the original datasets requires advanced statistical adjustments, with the feasibility and accuracy of such transportability efforts being an area of ongoing research [81].

### 3.11. TriNetX Strengths

Despite its limitations, TriNetX offers significant strengths that enhance research capabilities across various medical and healthcare fields. Topaloglu & Palchuk (2018) highlighted that TriNetX aggregates extensive real-world patient data from diverse healthcare institutions, enabling large-scale observational studies that provide insights beyond what is possible with randomized controlled trials[4]. The TriNetx platform facilitates rapid cohort identification, allowing researchers to define specific criteria and quickly identify patient cohorts, thereby streamlining the design and initiation of studies and accelerating evidence generation. Murad et al. (2018) emphasized that by connecting academic institutions, healthcare providers, and pharmaceutical companies, TriNetX fosters collaboration, advancing research across multidisciplinary teams [78]. Kennedy-Martin et al. (2015) pointed out that big database plays a critical role in generating real-world evidence for comparative effectiveness studies, health economics research, and post-marketing surveillance, complementing traditional trial data [82]. Leviton (2017) observed that it provides tools for propensity score matching and other statistical methods to minimize bias in observational research, enhancing the reliability of findings [80]. Ling et al. (2023) added that the platform’s data is regularly updated, offering near real-time insights, which is particularly valuable for monitoring emerging health trends or disease outbreaks [81]. Lastly, with a growing network of international contributors, TriNetX supports multinational studies, crucial for addressing global health challenges and understanding regional variations in care, underscoring its scalability for multinational research.

### 3.12. Balanced Insights

Recognizing both the strengths and limitations of platforms like TriNetX is crucial for guiding future research effectively. Topaloglu & Palchuk (2018) emphasize that acknowledging the real-world data scope of TriNetX can guide the development of more representative and practical study designs, leveraging its capabilities to include diverse populations and real-world conditions [4]. Murad et al. (2018) point out that recognizing issues such as selection bias and confounding factors is essential for implementing robust methodologies to mitigate these issues, thus enhancing the reliability of findings [78]. Understanding both strengths and limitations helps policymakers evaluate the applicability of findings for healthcare interventions, ensuring decisions are grounded in balanced evidence [1, 83]. Leviton (2017) highlights that the platform’s ability to produce real-world evidence is crucial for informing guidelines and improving clinical practice, supporting its integration into translational research [80]. Ling et al. (2023) state that recognizing advanced tools for data analysis encourages the continuous improvement and development of methodologies, such as AI-driven analytics, to maximize the platform’s utility [81]. Pearl & Bareinboim (2014) argue that awareness of both strengths and ethical challenges, such as data privacy, ensures responsible use and compliance with regulations, protecting patient rights while advancing research [79]. Finally, Chin et al. (2012) discuss how balancing the platform’s capabilities and limitations helps prioritize research areas where its strengths are most applicable, such as in rare disease studies or post-market drug surveillance [69].

### 3.13. Database Comparisons

Comparing TriNetX with other databases and research methods reveals both unique strengths and shared challenges. Topaloglu & Palchuk (2018) explain that, unlike centralized databases like SEER (Surveillance, Epidemiology, and End Results), TriNetX operates through a federated network model, facilitating multicenter data analysis while preserving data ownership at contributing sites, which reduces privacy risks but can introduce heterogeneity in data quality [4]. González et al. (2020) contrast TriNetX with the I2B2 platform, which requires more intensive efforts for semantic normalization and data integration, whereas TriNetX’s daily data refresh supports timelier research opportunities [53]. Singh et al. (2021) highlight that TriNetX supports enhanced clinical trial design by linking EMRs with pharmaceutical data, unlike systems like NSQIP (National Surgical Quality Improvement Program), which focus narrowly on surgical outcomes [3]. Hernandez et al. (2022) point out that TriNetX integrates genomic data using FHIR standards, enabling advanced pharmacogenomic and personalized medicine studies—a strength not commonly found in competing platforms [84]. Finally, Evans et al. (2021) observe that while TriNetX facilitates efficient multicenter collaborations, other federated systems like PCORnet face interoperability challenges due to variations in EMR platforms, highlighting different operational dynamics [23].

### 3.14. Limitation Mitigation

Minimizing the impact of limitations in future studies using TriNetX involves several strategic approaches. Topaloglu & Palchuk (2018) suggest standardizing data collection practices to ensure consistency in data entry and harmonization across participating healthcare organizations, which can reduce variability and improve dataset reliability [4]. Evans et al. (2021) recommend employing validation tools and monitoring data completeness, such as analyzing diagnosis-medication couplets to ensure robust medication data, to enhance data quality [23]. Evans et al. (2023) emphasize developing methodologies to detect and correct date-shifting practices in real-world datasets to ensure temporal accuracy and validity of clinical event timelines [85]. Antoine et al. (2023) advocate using advanced statistical methods, such as stabilized inverse-probability weighting and G-computation, to mitigate biases from confounding variables, thus improving causal inference [86]. Palchuk et al. (2023) highlight the importance of incorporating multilevel and longitudinal data analysis to better account for regional and temporal differences in healthcare practices, improving the generalizability of study findings [1]. Furler et al. (2012) note that expanding the TriNetX network to include more healthcare organizations from underserved and rural areas can improve the representativeness of its datasets[87]. Monjas et al. (2023) propose implementing automated validation and quality control mechanisms at the point of data entry to minimize errors and inconsistencies, enhancing the accuracy of datasets [88]. Lastly, Antoine et al. (2023) discussed the advantages of leveraging synthetic control arms in real-world evidence studies to enhance comparative effectiveness research by reducing reliance on incomplete or biased datasets [86].

### 3.15. Databases Comparison

Unlike the Surveillance, Epidemiology, and End Results (SEER) database, which maintains a centralized repository focused primarily on cancer-specific outcomes, TriNetX employs a federated network model that preserves data ownership at contributing sites while facilitating multicenter analysis. This architectural difference significantly impacts data access patterns and privacy management, with TriNetX offering more dynamic data updates compared to SEER’s annual reporting cycle [89].

When compared to PCORnet (Patient-Centered Clinical Research Network), TriNetX demonstrates different approaches to data standardization and interoperability. While PCORnet employs a common data model requiring extensive local data transformation, TriNetX’s approach to data harmonization allows for more rapid implementation across new sites. However, this efficiency comes with potential trade-offs in data granularity. PCORnet’s more rigorous standardization process may provide better consistency in certain research applications, particularly in longitudinal studies requiring detailed clinical parameters [90, 91].

The i2b2 (Informatics for Integrating Biology and the Bedside) platform presents another interesting comparison point. While i2b2 excels in providing detailed phenotypic data and supports sophisticated query building, it requires more intensive efforts for semantic normalization and data integration compared to TriNetX’s streamlined approach. TriNetX’s daily data refresh capability offers advantages for time-sensitive research, though i2b2’s more granular data model may be preferable for certain types of clinical research [92].

In terms of scalability, TriNetX has demonstrated superior capabilities in managing large-scale, multi-institutional studies compared to traditional research databases. Its integration of genomic data using FHIR standards enables advanced pharmacogenomic and personalized medicine studies, a functionality not commonly found in competing platforms [1, 4, 23, 84, 93–95]. However, this advantage must be weighed against the potential for data quality variations across participating institutions.

When reviewing data quality metrics, each platform presents distinct strengths and limitations. SEER’s rigorous data collection protocols ensure high-quality cancer-specific data but limit its utility for broader healthcare research. PCORnet’s emphasis on patient-centered outcomes provides rich patient-reported data but may face challenges in standardizing information across diverse healthcare settings. TriNetX’s approach to data quality focuses on rapid accessibility and broad coverage, though this may sometimes come at the expense of granular clinical detail [1, 4, 23, 84].

Usability comparisons reveal that TriNetX generally offers more intuitive interfaces for cohort identification and study design compared to traditional research databases. Its real-time query capabilities and integrated analytical tools provide advantages for rapid hypothesis testing and study feasibility assessment. However, platforms like i2b2 may offer more flexibility for complex phenotype definitions, albeit with a steeper learning curve [92].

## 4. Conclusions

The evaluation of TriNetX as a research platform reveals both significant strengths and notable limitations that researchers must carefully consider. While TriNetX offers unprecedented access to large-scale, real-world patient data and facilitates rapid cohort identification across multiple healthcare organizations, several critical limitations warrant attention. The platform’s reliance on EHR coding introduces potential inaccuracies, with studies indicating considerable variability in coding precision and completeness across institutions. Selection bias remains a significant concern, as the network predominantly represents insured patients from academic and acute care settings, potentially limiting generalizability to broader populations. These limitations are compounded by technological and practice disparities among participating healthcare organizations, which can affect data quality and representation. The transition between coding systems (ICD-9 to ICD-10) has introduced additional complexity, potentially impacting longitudinal studies and data consistency. However, TriNetX’s strengths in facilitating large-scale observational studies and supporting advanced statistical methods like propensity score matching partially mitigate these challenges. To enhance the platform’s utility, future developments should focus on standardizing data collection practices, expanding network representation to include more diverse healthcare settings, and implementing robust validation tools. Researchers should employ advanced statistical methods to address confounding variables and selection bias, while clearly acknowledging study limitations in their findings. The integration of automated validation mechanisms and quality control at the point of data entry could significantly improve data accuracy. Despite its limitations, TriNetX remains a valuable tool for medical research, particularly in areas such as rare disease studies, post-market surveillance, and comparative effectiveness research. Success in utilizing the platform requires a balanced approach that leverages its strengths while actively addressing its limitations through appropriate methodological choices and careful interpretation of results. Future enhancements should prioritize expanding network diversity, improving data validation mechanisms, and developing more sophisticated tools for bias mitigation.

## Data Availability

Data Availability Statement: This review article does not contain any new primary data. All information discussed is derived from previously published sources and publicly available databases, as cited in the manuscript.
